# Editorial: Medicinal *Cannabis*: evolution of therapeutic use, future approaches and other implications, volume II

**DOI:** 10.3389/fphar.2023.1322404

**Published:** 2023-11-13

**Authors:** Francesca Baratta, Shimon Ben-Shabat, Paola Brusa, Massimo Collino

**Affiliations:** ^1^ Department of Drug Science and Technology, University of Turin, Torino, Italy; ^2^ Department of Clinical Biochemistry and Pharmacology, Ben-Gurion University of the Negev, Be’er Sheva, Southern, Israel; ^3^ Department of Neuroscience “Rita Levi Montalcini”, University Hospital of the City of Health and Science of Turin, Turin, Toscana, Italy

**Keywords:** *Cannabis*, formulations, effectiveness, medical use, clinical trials

*Cannabis* has been historically used in the oldest traditional medicines. Nevertheless, in the last century, a negative vision has prevailed and *Cannabis* has for a long time been banned and declared illegal in many countries.

The recent marketing authorization of some products for medical use resulted in that *Cannabis*-derived products are gaining increasing attention. These compounds are emerging as potential treatments for a variety of medical conditions. Increasingly in recent years, scientific studies have contributed to provide a broader view of the different aspects related to the therapeutic use of cannabinoids.

Given the growing interest in medical *Cannabis*, the second volume of this Research Topic focused on the in-depth analysis of many aspects related to the medical use of *Cannabis*-based formulations, reporting original data and highlighting innovative perspective. For instance, Cairns et al. documented an increase in the prescription of *Cannabis*-based medicines in Australia for anxiety disorders, sleep-wake disorders, trauma- and stress-related disorders, and neurodevelopmental disorders, as well as for attention-deficit/hyperactivity disorder. The authors rightly underline that there is a dramatic lack of evidence-based clinical guidance on the use of *Cannabis*-derived products in psychiatry and, thus, most of the prescriptions are for pathologies for which there is no definitive clinical evidence. Besides, the high prevalence of prescribed THC (tetrahydrocannabinol)-containing products may rise concerns on their safety concerning (Cairns et al.). In fact, as documented by Stith et al. in real-time *Cannabis* consumption sessions, patients feeling “high” is often associated with improved symptom relief, but it also leads to dangerous increase in negative side effects.

Concerns regarding *Cannabis* side effects, legality and limited availability of information are also pointed out in the manuscript of Albert Garcia-Garcia-Romeu et al. who described the perceived advantages and challenges encountered by medicinal *Cannabis* users, concluding that the majority of participants reported benefits from *Cannabis* use for various conditions in the cases where conventional treatments were ineffective or undesirable (Garcia-Romeu et al.).

Rojas-Valverde and Fallas-Campos analysed the literature to investigate the use of CBD (cannabidiol) in athletes. CBD appears to have anti-inflammatory, neuroprotective, analgesic, anxiolytic and potentially recovery-inducing properties in athletes, but further scientific evidences are needed to confirm these effects. Furthermore, more consideration should be given to adopting a clearer and more comprehensive administrative policy for the use of *Cannabis* in sports (Rojas-Valverde and Fallas-Campos).

The use of non-psychoactive *Cannabis*-derived compounds such as CBD and CBG (cannabigerol) as chemotherapeutic agents requires further investigation. For this reason, the study conducted by Yüksel et al. explored the potential therapeutic synergy of a triple combination including CBD/CBG, curcumin and piperine in colon adenocarcinoma using HCT116 and HT29 cell lines. Curcumin and piperine have benn selected considering that clinical and epidemiological evidences, along with experimental results, suggest that these micronutrients may offer a safer approach to prevent tumour formation and its recurrence. The authors of this study demonstered a synergy in anti-tumorigenic effects between the investigated molecules (Yüksel et al.).

Del Rio et al. specifically directed their research on CBD as a potential therapy for fibrotic disorders. The antifibrotic effects of CBD in the skin were investigated *in vitro* and *in vivo* using NIH-3T3 fibroblasts, human dermal fibroblasts, and a bleomycin-induced skin fibrosis model. Moreover, non-alcoholic liver fibrosis was induced and investigated in mice. These experiments showed the potential role of the cannabinoid medicinal use in the management of fibrotic conditions, including systemic sclerosis and non-alcoholic fatty liver disease (Del Rio et al.).

Cajiao-Manrique et al. established a mouse model to investigate the neurobiological basis of cannabinoid addiction. In particular, they developed a model to study the neurobiological factors associated with resilience or susceptibility in the development of cannabinoid addiction. This model includes chemogenetic inhibition of neuronal activity in the pathway from the medial prefrontal cortex to the nucleus accumbens (Cajiao-Manrique et al.).

Regardless of the condition for which *Cannabis* extracts are intended to be used, it is essential to know their active molecule content. In this regard, Dei Cas et al. aimed to represent the Italian panorama of Cannabis oils, which were analysed to determine their cannabinoids content from 2017 to 2019. This study could be useful considering that the Italian law states that, in order to ensure the quality of the oil-based *Cannabis* preparation, the titration of the active substance(s) should be carried out. The quantification can be considered as the initial step for pharmacists to evaluate both the correct execution of preparation procedures and the quality of the extracts (Dei Cas et al.). In this field, also Pigliasco et al. developed their research taking into consideration the importance of developing suitable analytical methods useful to understanding the medicinal effects of *Cannabis*-derived products. Therefore a simple and rapid volumetric absorptive micro-sampling method combined with ultra-high-performance liquid chromatography coupled with mass spectrometry *in tandem* has been developed. This analytical method, which use a minimally invasive micro-sampling technique, could be useful for quantifying CBD, THC and their metabolites of relevant interest in patients with epilepsy treated with *Cannabis*-based preparations (Pigliasco et al.).

Overall, the articles included in the Research Topic offer new insights on mechanisms of action, potential risks and pharmacological properties of the components present in the *Cannabis* phytocomplex, confirming the significant interest clearly emerging in the evident potential of *Cannabis* in the medical field. At the same time, these findings confirm the need to further extend knowledge on the efficacy and safety profile of *Cannabis*-based preparations as well as in the development of suitable analytical methods to be applied in this field.

